# Renal and Vascular Effects of the Allosteric Transglutaminase 2 Modulator LDN-27219 in One-Kidney DOCA–Salt Mice

**DOI:** 10.3390/ijms26125724

**Published:** 2025-06-14

**Authors:** Ian Mees, Judit Prat-Duran, Simon Comerma-Steffensen, Ulf Simonsen, Estéfano Pinilla, Niels Henrik Buus

**Affiliations:** 1 Department of Biomedicine, Health, Aarhus University, 8000 Aarhus C, Denmark; ian_mees@web.de (I.M.); jprat@biomed.au.dk (J.P.-D.); simoncomerma@biomed.au.dk (S.C.-S.); us@biomed.au.dk (U.S.); estefanopinilla@biomed.au.dk (E.P.); 2 Department of Biomedical Sciences/Animal Physiology, Veterinary Sciences, Central University of Venezuela, Maracay 79CQ+VH3, Venezuela; 3 Department of Renal Medicine, Aarhus University Hospital, 8200 Aarhus N, Denmark

**Keywords:** salt, transglutaminase 2, albumin excretion, blood pressure, kidney fibrosis, vascular function

## Abstract

The enzyme transglutaminase 2 (TG2) has an open conformation with transamidase activity which crosslinks matrix proteins contributing to fibrosis development. LDN-27219 promotes the closed conformation of TG2, which can enhance vasodilation, but its effects in renal tissue are unknown. We investigated whether LDN-27219 treatment affects albuminuria and markers of renal fibrosis as well as ex vivo vasodilatation. Male C57BL/6 mice (n = 48) underwent unilateral nephrectomy plus insertion of a deoxycorticosterone acetate pellet (DOCA group) or nephrectomy only (sham group). Both groups were randomized to intraperitoneal treatment with either LDN-27219 (8 mg/kg twice daily) or vehicle for 2 weeks. Urine albumin excretion was evaluated by metabolic cages. Kidney tissue fibrosis markers were assessed by qPCR and Western blotting, while the TG2 conformational state was evaluated using native gel electrophoresis. Collagen staining was performed using Picrosirius red and quantified under circularly polarized light. Mesenteric arteries were mounted in wire myographs for evaluation of vasorelaxation. DOCA mouse developed significant albuminuria (*p* < 0.001 vs. sham), but neither TG2 mRNA nor protein expression was upregulated in the kidney. However, the relative amount of TG2 in the closed conformation was higher in DOCA mice. LDN-27219 did not affect albuminuria, but LDN-27219-treated DOCA mice showed less urine production and less collagen staining than vehicle-treated DOCA mice. LDN-27219 did not affect TG2 mRNA or TG2 protein expression or mRNA of fibrosis markers. LDN-27219-treated mice had enhanced vasorelaxation to the nitric oxide donor sodium nitroprusside. In conclusion, LDN-27219 treatment in the one-kidney DOCA–salt model did not affect renal TG2 mRNA and protein expression or albuminuria but still exerted beneficial effects in terms of reduced kidney fibrosis and urine production in addition to enhanced vasodilatation.

## 1. Introduction

**Figure 1:**
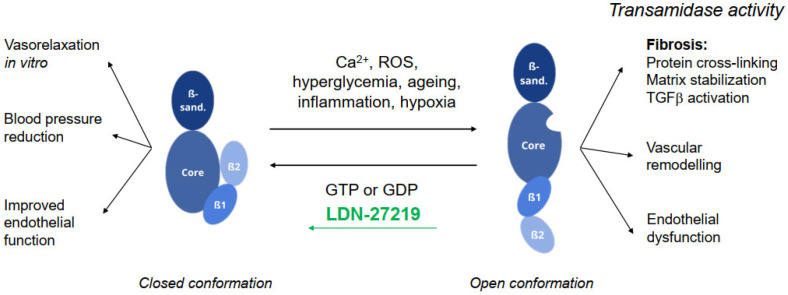
Illustration of the closed and open conformation of transglutaminase 2 (TG2) including stimuli able to induce a shift between the two states. ROS, reactive oxygen species. See text for further details.

Transglutaminases are ubiquitously expressed enzymes with numerous actions [1] among which the post-translational modification of proteins through the formation of iso-peptide bonds has attracted the most attention [2]. Transglutaminase 2 (TG2) is the most abundantly expressed member of the transglutaminase family, present in the extracellular matrix (ECM) as well as intracellularly [3,4]. All transglutaminases are characterized by a shared structure, consisting of four domains: the β-sandwich domain at the N-terminus, the α/β catalytic core domain, and two β-barrel domains at the C-terminus [5] (Figure 1). The calcium-binding feature of the enzyme causes it to adopt an open conformation, revealing the catalytic core and enabling it to carry out transamidase, esterification, and hydrolysis activities. A particular feature of TG2 is the ability to bind GTP/GDP on a specific site located between the catalytic core and the first β-barrel domain [6,7]. The binding of GTP/GDP promotes the movement of the β-barrel domains, making the enzyme shift into a compact conformation, also named the closed conformation (Figure 1) [8]. Despite being in the closed conformation, TG2 can bind calcium ions even with GTP present, but a high calcium concentration is necessary to induce a conformational shift [9]. Thus, the closed conformation will inhibit the open conformation activities [6]. However, the closed conformation of TG2 is not inactive since it presents GTPase and protein kinase activities [6] by hydrolyzing GTP and ATP, respectively [10].

Extracellularly, the open conformation is involved in the Ca^2+^-dependent crosslinking of both glutamine with lysine residues, following the prior deamination of glutamine [3,4]. This results in protein crosslinking, leading to extracellular matrix stabilization and thus contributing to fibrosis development [11]. Furthermore, TG2 crosslinking is involved in transforming growth factor beta (TGFβ) release and activation, which exacerbates fibrosis [12]. In addition, TG2 transamidase activity has been associated with vascular remodeling [13] and endothelial dysfunction [14]. In this regard, the transamidase activity of TG2 is considered a sign of cellular stress, and stimuli like increased extracellular glucose levels [15], reactive oxygen species, and inflammatory cytokines [14] have been shown to increase it.

The closed conformation of TG2, on the other hand, is important for regulating the vascular tone through actions on membrane ion channels [3,4,16]. The compound LDN-27219 (2-[(3,4-dihydro-4-oxo-3,5-diphenylthieno[2,3-d]pyrimidin-2-yl)thio]acetic acid hydrazide) is a non-competitive reversible modulator of TG2 that docks to a site related to the GTP-binding pocket of TG2 [17], stabilizing the enzyme in its closed conformation [18] (Figure 1). In a recent study, we found this compound to potentiate nitric oxide (NO) dependent vasorelaxation in vitro through the opening of potassium channels and to induce acute blood pressure (BP) reductions following intravenous infusions in anesthetized rats [12].

Various animal models of kidney damage have shown TG2 to be involved in renal fibrosis development [19,20,21], and the knock-out of TG2 is associated with slower fibrosis development following unilateral ureteral obstruction [22,23]. Pharmacological inhibition of TG2 has been attempted with various drugs [20]. Nonetheless, the used inhibitors tend to lock TG2 in its open conformation, reducing transamidase activity as well as the closed conformation activities, which could prevent the positive cardiovascular effects of the closed conformation. We recently demonstrated that LDN-27219 is able to reduce the renal mRNA expression of profibrotic markers in kidneys subjected to ureteral obstruction [24].

Treatment of mice and rats with deoxycorticosterone acetate (DOCA), in combination with increased dietary salt, is considered a model of salt-sensitive hypertension mainly due to hypervolemia, sympathetic nervous overactivity, and central neurogenic effects [25,26,27,28]. Together with the reduction in the nephron mass, this model can also result in elevated urinary albumin excretion [22,29] and, in some studies, the development of renal fibrosis [22,25,30,31]. Urinary protein and albumin excretion are considered early and clinically important markers of kidney damage, but albuminuria levels are only reported in a few studies concerning TG2 inhibition [20,32].

In the present study, we hypothesized that LDN-27219 treatment would improve albuminuria and markers of renal fibrosis in the one-kidney DOCA–salt model. To address the hypothesis, we investigated the renal effects of 2 weeks of LDN-27219 treatment in terms of albumin excretion and renal TG2 gene expression, protein expression, TG2 conformation, and transamidase activity in addition to fibrosis development. Furthermore, we included investigations on isolated small arteries ex vivo to establish possible vascular effects of the treatment.

## 2. Results

### 2.1. Animals

**Figure 2:**
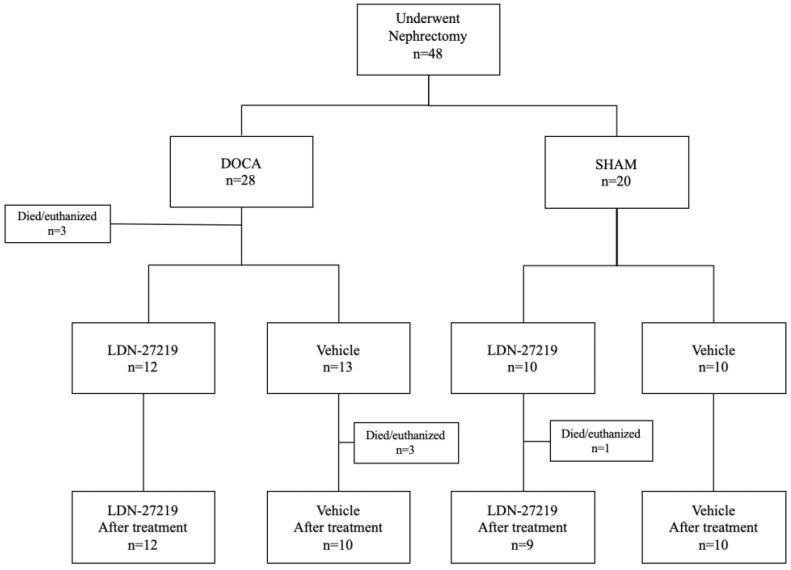
Flowchart of the experiment. Randomization of C57/BL6 mice into DOCA and sham groups and into treatment groups with LDN-27219 or vehicle. Three mice in the DOCA group were euthanized after surgery but before the start of treatment. Three mice died during the treatment period in the DOCA vehicle group, while one mouse died during the treatment period in the sham LDN-27219 group.

A flow chart describing the fate of the 48 mice subjected to nephrectomy is shown in Figure 2. In the DOCA group, three mice died before the start of treatment. Three mice from the DOCA vehicle arm were euthanized or found dead during treatment. In the sham group, one mouse in the LDN-27219 arm died during the treatment period.

There were no differences in body weight at baseline or during the experiment between the four groups. Organ weights (adjusted for body weight) of the heart, the liver, the remaining kidney, and the spleen were higher in the DOCA mice than in the sham animals. LDN-27219 treatment did not significantly affect organ weights (Table 1).

### 2.2. Kidney Function and Blood Pressure

**Figure 3:**
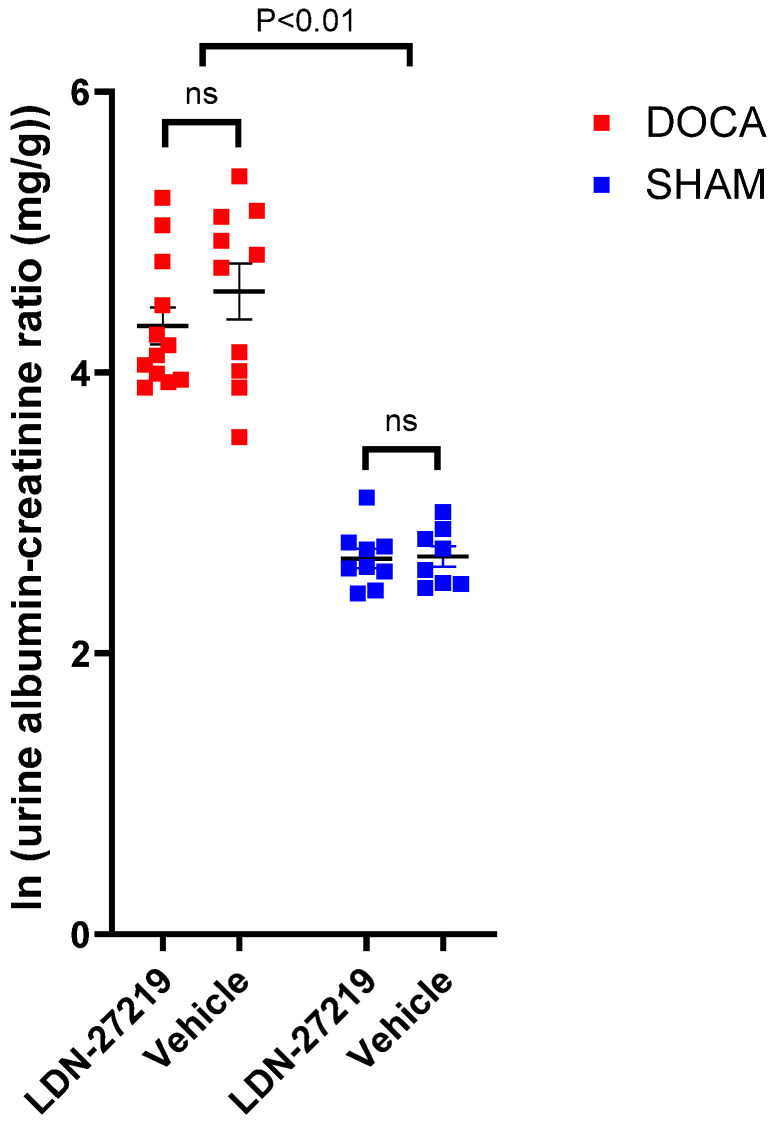
Urine albumin/creatine ratio (logarithmically transformed) for DOCA mice treated with LDN-27219 (n = 12) or vehicle (n = 10) and sham mice treated with LDN-27219 (n = 9) or vehicle (n = 10). Data were analyzed with 2-way ANOVA and are expressed as mean ± SEM. ns = non significant.

By the end of the 2-week treatment period, DOCA mice had a much higher 24 h urine output than the sham mice (Table 1), but it was lower in the LDN-27219-treated as compared to the vehicle-treated DOCA mice. Plasma urea levels were significantly lower in DOCA mice than in sham mice but not affected by the LDN-27219 treatment. The DOCA animals developed significant albuminuria, which, however, was unaffected by LDN-27219 treatment (Figure 3). DOCA did not result in a significant increase in MAP, and we did not detect an effect of LDN-27219 on MAP (Table 1).

### 2.3. Renal Fibrosis

**Figure 4:**
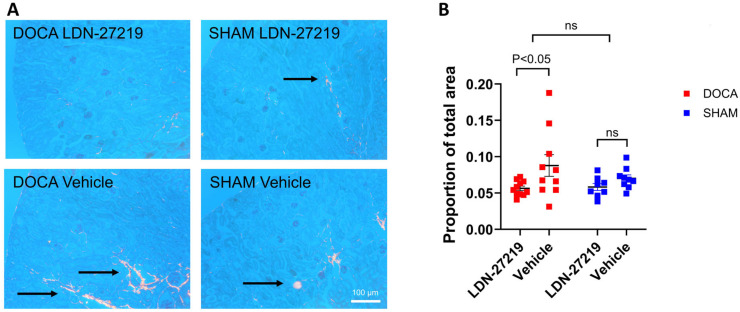
(**A**) Representative images of the Picrosirius red collagen staining (arrows) visualized under circularly polarized light from the different groups with (**B**) corresponding individual and mean quantification of positive picrosirius red staining percentage areas. Data were analyzed with 2-way ANOVA and are expressed as mean ± SEM. ns = non significant.

Figure 4A shows representative examples of Picrosirius staining. Although there was no overall difference between DOCA and sham groups concerning the degree of Picrosirius red staining, we observed a lower collagen deposition in the LDN-27219-treated as compared to vehicle-treated in the DOCA mice (Figure 4B).

### 2.4. TG2 mRNA, Protein Expression, TG2 Conformation and Transamidase Activity

**Figure 5:**
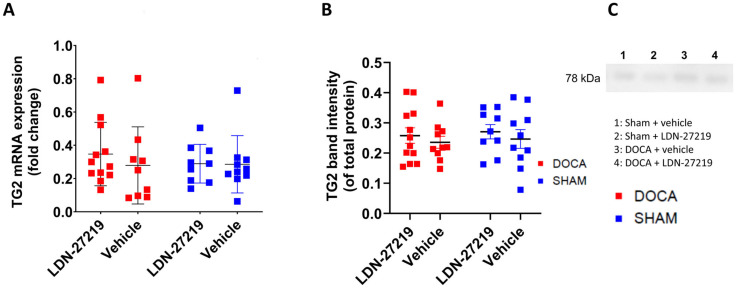
(**A**) Kidney TG2 mRNA expression based on qPCR and (**B**) averaged kidney TG2 protein expression (TG2/total protein) based on Western blotting in DOCA, and sham mice treated with LDN-27219 or vehicle. (**C**) Representative blots. The 2-way ANOVA was non-significant for both TG2 mRNA and TG2 protein. Data show individual results and mean ± SEM.

We found no difference in TG2 mRNA expression (Figure 5A) or protein expression (Figure 5B) between DOCA and sham animals, and LDN-27219 had no effect on these parameters. Figure 5C shows representative Western blot examples.

Supplementary Figure S1 shows the original TG2 Western blots, and Supplementary Figure S2 shows blots including the positive control with mTG2. Supplementary Figure S3 depicts total protein quantification from TG2, BPA, and fibronectin (FN) Western blots.

Individual Ct values are given in Supplementary Table S3. The low variation in Cq values, seen in Supplementary Figure S4, documents the stability of GADPH as a housekeeping gene.

TG2 transamidase activity also showed no difference between the groups, and the LDN-27219 treatment did not influence it (Supplementary Figure S5), with the original blots shown in Supplementary Figure S6. However, due to BPA being cell-impermeable, we evaluated the penetration of BPA into the kidney tissue through staining (Supplementary Figure S7), which revealed a very low penetration limited to the outer part of the cortex.

**Figure 6:**
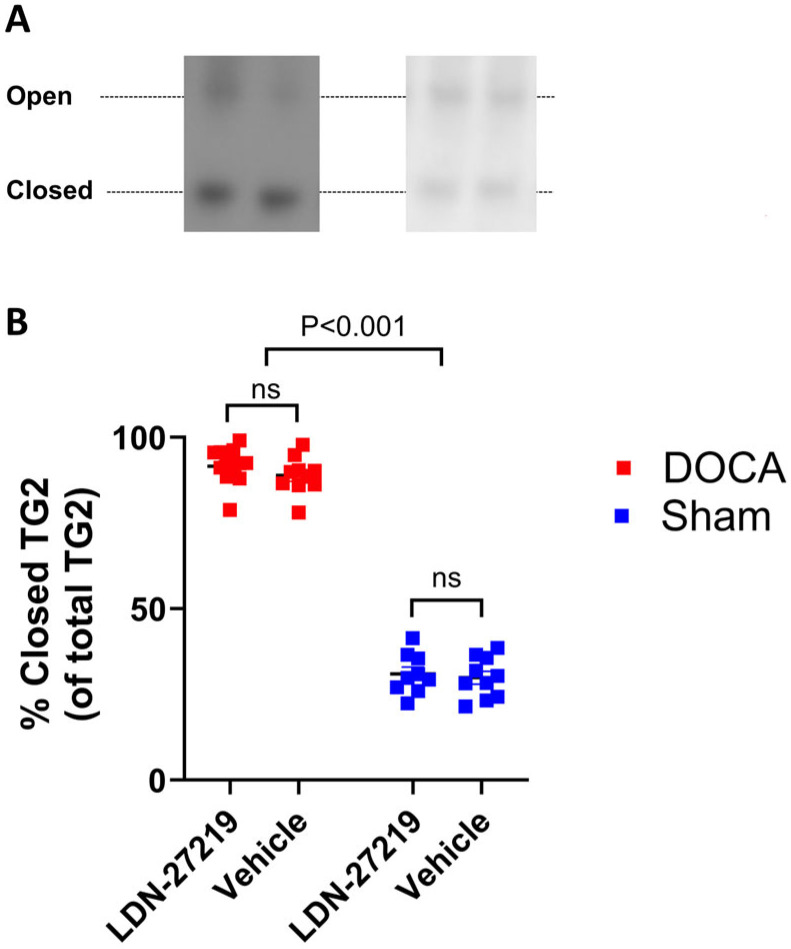
(**A**) Native PAGE with representative blots showing two bands corresponding to the open and closed conformation of TG2 and (**B**) average percentage of kidney TG2 protein present in the closed formation in DOCA mice treated with LDN-27219 (n = 12) or vehicle (n = 10) and in sham mice treated with LDN-27219 (n = 9) or vehicle (n = 10). Data were analyzed with 2-way ANOVA and are expressed as mean ± SEM. ns = non significant.

We then assessed the conformational state of TG2 in the renal tissue to examine whether it shifted to an open state under the influence of the DOCA. However, DOCA mice showed a larger proportion of TG2 in the closed form than the sham mice (Figure 6), and we found no influence of LDN-27219 treatment on the distribution between the closed and open forms of TG2. The original native blots are shown in Supplementary Figure S8.

**Figure 7:**
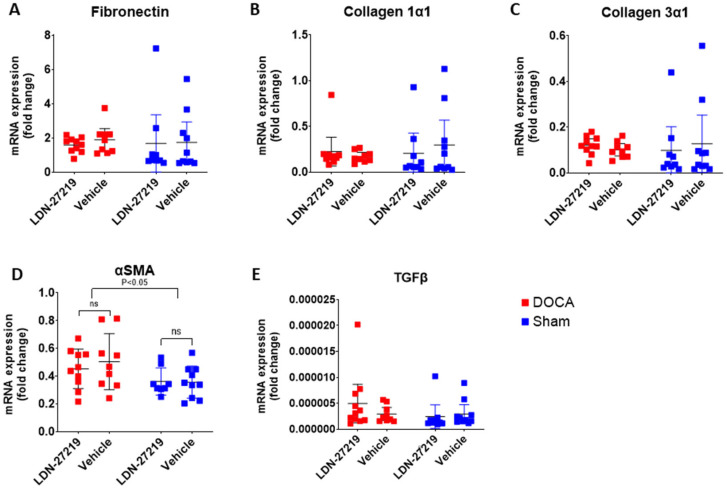
Renal mRNA expression based on qPCR for (**A**) fibronectin, (**B**) collagen 1α1, (**C**) collagen 3α1, (**D**) α–smooth muscle actin (αSMA) and (**E**) transforming growth factor beta (TGFβ) in DOCA mice treated with LDN-27219 (n = 12) or vehicle (n = 10) and in sham mice treated with LDN-27219 (n = 9) or vehicle (n = 10). The 2-way ANOVA was only significant for αSMA. Data show individual values and mean ± SEM. ns = non significant.

The qPCR data (Figure 7A–E) showed elevated α-smooth muscle actin in DOCA mice, but none of the other fibrosis markers, fibronectin, collagen 1α1, collagen 3α1, or TGFβ, differed between DOCA and sham mice. There were no effects of LDN-27219 treatment on any of these markers. Individual Ct values can be found in Supplementary Table S3. In addition to qPCR, Western blotting was performed for renal fibronectin expression, but this protein was not affected by either DOCA or LDN-27219 (Supplementary Figures S9 and S10).

### 2.5. Vascular Function

**Figure 8:**
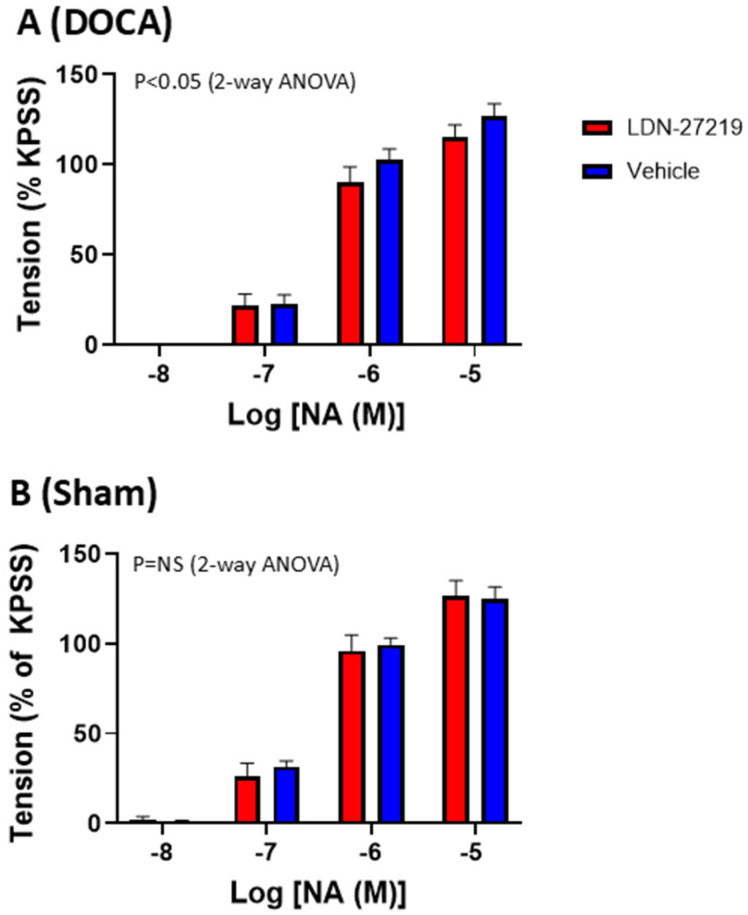
(**A**) Average constriction of mesenteric small arteries to noradrenaline (NA) in DOCA mice treated with LDN-27219 (n = 12) or vehicle (n = 10) and (**B**) average constriction to NA in sham mice treated with LDN-27219 (n = 9) or vehicle (n = 10). The responses are normalized to the tension obtained with KPSS. Data are mean ± SEM.

**Figure 9:**
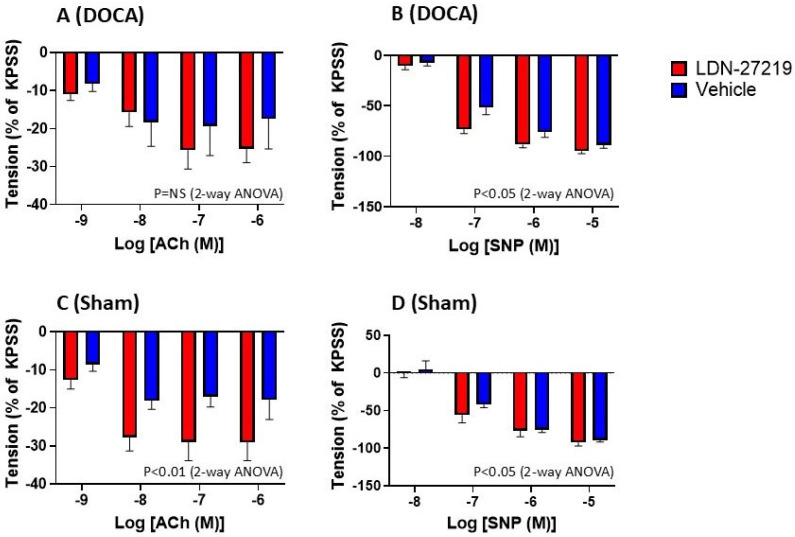
Mesenteric resistance artery relaxation to acetylcholine (ACh) and sodium nitropruside (SNP) following pre-constriction of the vessels with phenylephrine to achieve a tension corresponding to 60–80% of that induced by KPSS. Responses were normalized to the tension obtained with KPSS. The upper panel (**A**,**B**) shows data for DOCA mice treated with LDN-27219 (n = 12) or vehicle (n = 10), and the lower panel (**C**,**D**) shows data for sham mice treated with LDN-27219 (n = 9) or vehicle (n = 10). Data are mean ± SEM.

The internal diameters and force development of the mesenteric arteries are shown in Table 2. There were no differences in vessel diameters or pre-constriction levels between the groups. The results of the constrictions to NA are shown in Figure 8. LDN-27219 treatment slightly attenuated constrictions to NA in DOCA mice (*p* < 0.05) but not in sham mice. Figure 9 illustrates relaxation to ACh and SNP. LDN-27219 treatment increased relaxations to ACh, although this was only significant in sham mice (*p* < 0.01). LDN-27219 treatment enhanced the relaxation of SNP in both DOCA and sham mice (*p* < 0.05).

## 3. Discussion

The present study, which used a mouse one-kidney DOCA model, could not detect any effects of the TG2 modulator LDN-27219 on albuminuria, but its administration did result in reduced collagen deposition and urine production, indicating a potential renal effect. In addition, isolated small arteries from LDN-27219-treated animals showed improved relaxations to SNP.

### 3.1. The One-Kidney DOCA Model

Uni-nephrectomized DOCA–salt-treated mice are characterized by hyperfiltration, increased sodium excretion, and diuresis, as well as increased kidney weight [29,33,34]. This was also evident in our DOCA animals, which had a more than 5-fold increase in urine volume and more than 20% increase in kidney weight compared to their sham counterparts. We did not detect differences in body weight between DOCA and sham mice but found higher wet weights of both the liver and the spleen, which suggests some degree of fluid congestion.

The C57BL/6 mouse strain may not respond to DOCA in terms of a significant BP increase, despite apparent renal effects with albuminuria and an increase in cardiac mass [35,36]. This has been explained by their genetic background, with only one renin gene and a low baseline renin level [27,35]. Also, in our study, the DOCA mice, on average, remained normotensive throughout the treatment period. Although we used a standard tail-cuff method for BP measurements, this may have larger variability than telemetric BP recordings and cannot detect smaller differences in MAP. Furthermore, our protocol included twice daily intraperitoneal injections, with a risk of a stress response in both DOCA and sham mice, also masking minor variations in BP between groups. The observed resistance to DOCA, as concerns the development of high BP, may limit the usefulness of the C57BL/6 strain as a model of salt-sensitive hypertension in humans.

### 3.2. LDN-27219

LDN-27219 is a lipophilic, cell-permeable acyl hydrazide derivative that reversibly blocks TG2 transamidase activity, mimicking the inhibitory effect of GTP [37,38]. This results in a change to the closed formation [17]. The pharmacokinetics of LDN-27219 have previously been characterized following a single intraperitoneal administration of 2 mg/kg in C57BL/6 mice [12]. This revealed a systemic bioavailability of 65% and a half-life of approximately 5 h, and in the present study, we chose a dose of 8 mg/kg every 12 h [24]. Our data showed comparable weights and survival among LDN-27219 and vehicle-treated animals, which supports good tolerability of the drug.

### 3.3. Albuminuria, Renal Fibrosis, and Fibrosis Markers

The occurrence of albuminuria in our DOCA-treated C57BL/6 mice is in accordance with previous studies using the same strain [22,39]. We could not detect an apparent increase in the production or amount of TGFβ or fibrosis markers using qPCR and Western blotting. The Picrosirius red staining under polarized light is considered the standard for quantifying collagen deposition. With this method, LDN-27219-treated DOCA mice showed a reduction in collagen compared to the DOCA controls. Still, the vehicle-treated mice did not show increased collagen deposition, and the relative area of fibrosis was, on average, low. The lack of marked increases in fibrosis biomarkers synthesis or histologically detectable fibrosis development may be related to the absence of hypertension or be explained by a too-short DOCA exposure. Although LDN-27219 showed signs of antifibrotic potential, these findings demonstrate albuminuria as a much earlier phenomenon than renal fibrosis development. The limited development of fibrosis in DOCA mice calls for experiments of a longer duration to understand the extent of the antifibrotic properties of LDN-27219.

Additionally, we observed a lower urine volume in the LDN-27219-treated DOCA mice. This suggests a functional effect of LDN-27219, but whether this is primarily due to an impact on the kidney or less water consumption in the animals is unknown.

### 3.4. TG2 Transamidase Activity and Conformation

Our model did not show an increased kidney transamidase activity, and kidneys from animals treated with LDN-27219 presented similar levels of BPA incorporation in the absence of LDN-27219. TG2 transamidase activity has always been studied through the indirect measurement of an incorporated substrate [20]. We chose to evaluate the incorporation of BPA in intact kidney tissue as we wanted to maintain the natural compartmentalization of TG2 in the kidney, instead of using homogenized samples. However, BPA is cell-impermeable, which is probably responsible for its low penetration into the tissue, leading to a potential misinterpretation of the activity levels. The difference in TG2 conformation distribution between DOCA and sham kidneys further supports this. While sham kidneys showed TG2 in both conformations, DOCA kidneys showed an increased presence of closed TG2. The reason for this is still unknown, but it is in agreement with our observations in kidneys from mice subjected to unilateral ureteral obstruction (UUO) [24]. In UUO mice, TG2 transamidase activity levels were increased when studied in thin slices, while protein levels were decreased, suggesting a faster TG2 protein turnover. Moreover, with investigations showing that closed conformation prevents TG2 protein degradation [40,41], the conformational assay would only show the closed form, while TG2 in the open conformation is lost. Thus, our conformational study of TG2 supports the effect of DOCA on TG2, likely activating its transamidase activity and undergoing protein degradation.

### 3.5. Vascular Effects of LDN-27219 Treatment

In the present study, we did not notice significant differences between DOCA and sham mice concerning contraction or relaxation of mesenteric arteries, possibly associated with the lack of hypertension development in the mice. Still, the occurrence of albuminuria suggests capillary alterations in the renal vasculature, but a systemic vascular effect of DOCA could not be documented in this 3-week protocol.

In a recent study, we described the direct vasodilatory effects of LDN-27219 and improvement in both endothelium-dependent and -independent relaxations in isolated resistance arteries from rats and humans [12]. This effect is mediated by increased sensitivity of the smooth muscle layer to NO through the opening of large-conductance calcium-activated potassium channels (BKCa) by the closed conformation of TG2. The present data from mice isolated arteries subjected to in vivo treatment with LDN-27219 support these findings. Our experiments were conducted at least 12 h following the last drug administration to the animal, and we expect the drug to be washed out of the preparation before the construction of the concentration-response curves. The enhanced relaxation to SNP could therefore point to an increased expression or activity of BKCa induced by the preceding 2-week LDN-27219 treatment. The slightly attenuated constriction to NA in the LDN-27219-treated DOCA mice aligns with this. Taken together, LDN-27219 treatment has significant vascular effects in terms of increased NO-dependent relaxation.

## 4. Methods

### 4.1. Animals

Male C57BL/6 mice (Taconic, Ry, Denmark) arrived at the animal facility at 7–8 weeks of age and were housed in cages in groups of 3 but were split up if showing signs of aggressive behavior. The mice had free access to water and standard chow (Altromin 1328, Brogaarden, Denmark) and were kept under a day–night cycle of 12 h. The animals were under close monitoring, with weight measurement twice weekly and systematic scoring of welfare (Supplementary Table S1) every 4 days. Animals below a pre-defined welfare score were euthanized. The National Danish Animal Research Inspectorate approved the project (approval number: 2020-15-0201-00617), and the persons involved in handling the animals had the required license. The Animal Research: Reporting of In Vivo Experiments (ARRIVE) guidelines are followed in this work [42].

The personnel involved in handling and treating the mice, measuring and analyzing outcomes, and interpreting the results were blinded to the treatment regimen.

### 4.2. Blood Pressure Measurement

After an acclimatization period of at least 2 days, BP measurements were initiated using tail-cuff equipment (Kent Scientific, Torrington, CT, USA) with a corresponding heat plate. To accustom the mice to the procedure, they were trained to be placed into the fixating plastic tube and covered with a drape one week before the measurements started. During the BP measurement, a heating lamp was kept 30–40 cm above the drape to maintain a constant temperature (monitored by a thermometer) and ensure blood flow to the tail. Twenty measurement cycles were performed, the first 10 of which were discarded. BP data from the remaining 10 cycles were averaged after the exclusion of values more than 2 standard deviations from the mean using Rstudio (version 1.3.1073).

### 4.3. Nephrectomy and DOCA Pellet Insertion

The mice were randomized by lot drawings in groups of 3 to undergo either unilateral nephrectomy plus insertion of a DOCA pellet or nephrectomy alone (sham). All mice were anesthetized with the induction of 5% isoflurane in atmospheric air, followed by subcutaneous injections of 0.17 mL buprenorphine (Temgesic^®^, Eumedica S.A., Manage, Belgium 0.015 mg/mL in saline) and 5 mg/kg carprofen (Norodyl^®^, Norbrook Laboratories Limited, Newry Co. Down, Northern Ireland, 0.05 mg/mL in saline). After anesthesia induction, the animal was placed on a heating plate for temperature control. Anesthesia deepness was defined as the absence of pedal withdrawal reflex and was maintained using continuous isoflurane 2–3% in atmospheric air.

The right flank was prepared with removing hair and disinfection with 0.2% chlorhexidine with the animal in the ventral position. The skin and muscle layer were opened, the renal pelvis vessels and ureter were ligated using reabsorbing 3-0 or 4-0 vicryl, and the kidney was removed together with its capsule and adrenal gland. Then, the muscle layers and skin were closed separately by 6-0 silk sutures. A DOCA pellet (50 mg, with a release time of 21 days Innovative Research, Sarasota, FL, USA) was inserted in the left upper side of the back using the incision from the nephrectomy after blunt dissection of the subcutis to the left side of the back. A 6-0 silk suture was placed between the fascia and skin to minimize pellet movement. The skin was sutured with 6-0 silk. Sham mice had the same procedure except for the insertion of a pellet.

After the termination of anesthesia, the animals received 100% oxygen on a mask for one minute and were monitored for the next hours. The mice received daily subcutaneous injections of carprofen (5 mg/kg/day) for three days following the surgical procedure.

### 4.4. Treatment

Seven days after the nephrectomy, the mice were randomized to receive treatment with either LDN-27219 or vehicle. LDN-27219 powder (Tocris Bioscience, Bristol, UK) was dissolved in polyethylene glycol 400 (PEG-400) 100% to a stock solution of 20 mg/mL and frozen at −21 °C. The stock solution was thawed and dissolved in 15% sulfobutyl ether beta-cyclodextrin sodium (SBE-b-CD, Glentham Life Science, Wiltshire, UK) in 0.9% saline right before use, reaching a final concentration of 1 mg/mL LDN-27219. LDN-27219-treated mice received a dose of 8 mL/kg body weight, and vehicle-treated mice received the same volume (5% PEG-400 in 15%SBE-b-CD/0.9% saline) twice daily for 2 weeks. The injections were performed intraperitoneally with 27 or 30 G needles and spaced for 12 h.

After nephrectomy, sham mice continued with tap water, while DOCA mice were switched to ad libitum saline water (0.9% NaCl and 0.2% KCl in tap water) for the remaining part of the experiment.

### 4.5. Sample Collection

For the last 24 h of the 2-week treatment period, each animal was kept solitary in a metabolic cage to collect urine, with the mice having access to unlimited drinking water and chow. The urine samples were stored at −18 °C and analyzed en bloc for creatinine and albumin concentrations.

By the end of treatment, the mice were anesthetized with an intraperitoneal injection of 0.17 mL 80 mg/kg pentobarbital in 1 mg/mL lidocaine, which was repeated if the pedal withdrawal reflex was still present after 10 min. The abdomen and the rib cage around the sternum were then opened, and blood was drawn from the heart with a syringe and 23 G needle and immediately transferred into lithium heparin-coated tubes. Afterward, blood was transferred to Eppendorf tubes to be centrifuged at a relative centrifugal force of 4573× *g* while being cooled to 4 °C, and the plasma samples were frozen at −80 °C. To ensure a sufficient 200–300 µL volume, it was necessary to pool plasma from 2–3 animals from the same treatment arm. The samples were analyzed en bloc for urea concentrations at the Department of Clinical Biochemistry, Aarhus University Hospital.

The remaining kidney was dissected, and the capsule was removed. A needle biopsy (2 mm in diameter) from the upper pole was performedand stored for qPCR analysis (see Section 4.8).

### 4.6. Transamidase Activity Assay

Immediately thereafter, the kidney was incubated for 4 h at 37 °C in 1 mM biotinamide pentylamine (BPA) (21345, ThermoFisher Scientific, Waltham, MA, USA) diluted in physiological saline solution (PSS) with the following composition (mmol/L): 119 NaCl, 4.7 KCl, 1.18 KH_2_PO_4_, 1.17 MgSO_4_, 1.5 CaCl_2_, 24.9 NaHCO_3_, 0.026 ethylene diamine tetra acetate (EDTA), and 5.5 glucose. During the incubation, the samples were continuously bubbled with a gas mixture of 75% N_2_, 20% O_2_, and 5% CO_2_. Later, unincorporated BPA was rinsed by washing the kidney 3 times in Dulbecco’s phosphate-buffered saline (PBS). The kidney was then cut in two halves; the upper part was snap-frozen and stored at −80 °C for further protein analysis (see Section 4.9). The lower part was fixed in formaldehyde for 24 h at 4 °C and changed into 70% ethanol until embedded in paraffin (see Section 4.7).

Incorporated BPA, as a proxy for transamidase activity, was detected on a Western blot using streptavidin–HRP in homogenized samples and through immunofluorescence using streptavidin conjugated to Alexa Fluor 594 in the fixed samples.

### 4.7. Renal Histology

Kidney tissue embedded in paraffin was sliced at 5 µm thickness and stored until further use. Kidney sections were then dried at 55 °C and rehydrated with xylene and ethanol. Picrosirius red staining solution (ab150681, Abcam, Cambridge, UK) was added to the sections for 20 min and then dehydrated and mounted in Eukitt Quick-Hardening Mounting Media (03989, Sigma-Aldrich, St. Louis, MO, USA). Five fields of view at 20× were used for each kidney. These 5 images covered an axial slice of the entire kidney, except for the pelvis, which is irrelevant for assessing fibrosis. Collagen fibers were visualized in a circularly polarized light microscope. Data are expressed as the area of collagen stained versus total kidney area, quantified using the free software ImageJ2 (Fiji, v2.16.0).

Deparaffinated slices were also permeabilized and blocked with 2% bovine serum albumin, 0.5% Tween-20, and 0.1% Triton X-100 in Tris-buffered saline. Then, the slices were incubated with streptavidin Alexa Fluor 594 conjugate (1:100; S11227, ThermoFischer Scientific) and counterstained with DAPI. The slices were then mounted in GlycerGel (C056330-2, Agilent, Santa Clara, CA, USA) containing 2.5 *w*/*v*% 1,4-Diazabicyclo[2.2.2]octane. The imaging of the slices was conducted using an EVOS microscope at the 4× objective.

### 4.8. qPCR of Renal Tissue Samples

RNA was isolated from the kidney biopsy using the NucleoSpin RNA Plus kit (740984.250, Macherey—Nagel, Dueren, Germany). RNA concentration was measured with the NanoDrop, and cDNA was synthesized using Reverse Transcriptase SuperScrip IV (18090050, Invitrogen). Primers and probes for different targets were designed and validated (Supplementary Table S2). qPCR was performed in the AriaMx Real-time PCR system (Agilent Technologies, Santa Clara, CA, USA) in a 50-cycle protocol. We analyzed outliers using the Robust OUTlier removal tool in GraphPad Prism V. 8.4.3 (ROUT method). The target Ct values were then normalized against Ct values obtained with GAPDH and expressed as fold change of the housekeeping gene using the following formula: (1/2^∆Ct^) × 100. Data presentation is in accordance with guidelines [43].

We validated the GAPDH specificity of our primers by sequencing the resulting amplicon (Eurofins Genomics Europe Shared Services GmbH, Ebersberg, Germany). Then, the sequencing results were blasted using Blastn suite 2.17.0+ using the mouse genomic plus transcript database. All transcript variants of GADPH were detected, and only some non-coding RNA from predicted genes or pseudogenes were significantly aligned with a lower percentage of identity.

### 4.9. Western Blotting

The kidney tissue was homogenized in RIPA lysis buffer (0.5 mmol/L tris/HCL pH 7.4, 10 mmol/L EDTA, 1.5 mmol/L NaCl, 2.5% deoxycholic acid, 10% NP-40) with Halt Protease and Phosphatase Inhibitor Cocktail (78441, ThermoFisher Scientific) and disrupted mechanically. Later, samples were sonicated and centrifuged at a relative centrifugal force of 19,817 g at 4 °C for 10 min. The supernatant was collected, and the protein was quantified using Lowry’s method. Samples for running were prepared at a final concentration of 2 µg/µL mixed with 2× Laemmli sample buffer (1610737, Bio-Rad, Hercules, CA, USA) and loaded on Criterion TGX Stain-Free Gel 4–15% (5678085, Bio-Rad) and transferred onto PVDF membranes. The total protein was read after transferring by exposing the membrane to UV light. For the detection of transamidase activity, the membrane was blocked in BSA 5% and incubated in streptavidin–HRP conjugate (1:10,000; RPN1231, Cytiva Amersham, Amersham, UK) for 1 h at room temperature and subsequently developed with ECL Prime Western Blotting System (RPN2232, Cytiva Amersham, Amersham, UK). TG2 and fibronectin (FN) membranes were blocked in EveryBlot Blocking Buffer (12010020, Bio-Rad) for 5 min and incubated with the primary antibodies rabbit anti-TG2 (1:500; ab421, Abcam, Cambridge, United Kingdom) and rabbit anti-fibronectin (1:1000; ab2413, Abcam), respectively. Finally, membranes were incubated with goat anti-rabbit (1:4000; G-21234, Invitrogen) and developed with ECL Prime Western Blotting System (RPN2232, Cytiva). The membranes were then read in the PXi 4 Touch (Syngene, Bangalore, India). The bands were quantified using the GeneTools program, and an intermembrane normalization was performed using a pooled sample. Later, the samples were normalized again using total protein, accounting for loading defaults. To ensure we only analyzed the band corresponding to TG2, we validated the band beforehand using recombinant mTG2.

### 4.10. Assessment of TG2 Conformation

Homogenized kidney samples were prepared by mixing 1:1 with native sample buffer (1610738, Bio-Rad). Then, 15 µg of the sample was loaded in Triton Stain-Free Gels 4–15% (5678085, Bio-Rad) and run at 125 V for 1 h and 15 min in Tris/Glycine buffer (1610771, Bio-Rad) on ice. After running, the protein was transferred as previously described and immunoprobed with anti-transglutaminase 2 (1:1000; A072, Zedira, Darmstadt, Germany). The two resulting bands, corresponding to the open and closed conformation, were analyzed as the percentage of total TG2.

### 4.11. Vascular Function

After euthanasia, the mesenteric bed was removed and placed in cold (4 °C) PSS. Small arteries were carefully dissected, from the 2nd or 3rd branch (200–300 μm in diameter), cut into lengths of 1–2 mm, and mounted on two 25 μm tungsten wires in small vessel myographs (Danish Myo Technology, Hinnerup, Denmark). After mounting, the vessel chamber was heated to 37 °C and continuously bubbled with gas (75% N_2_, 20% O_2_, and 5% CO_2_). The arteries were calibrated stepwise, increasing the vessel diameter to find the optimal diameter for isometric tension recordings, which corresponded to 0.9 times the estimated internal diameter at 100 mmHg of transmural pressure.

After calibration, the arteries were left for at least 20 min, after which they were contracted twice for 2 min with PSS containing 124 mM of KCl (KPSS, where KCl substituted NaCl on an equimolar basis). Three concentration-response curves were then constructed for acetylcholine (ACh), noradrenaline (NA), and sodium nitroprusside (SNP). Vasorelaxations to ACh and SNP were performed after contraction of the vessels with phenylephrine to achieve a tension corresponding to 60–80% of that induced by KPSS. ACh, NA, and SNP were diluted from a stock solution in demineralized water. Only data from vessel segments developing a tension of 2 mN or more to the KPSS were included. All myograph recordings were carried out via Labchart V and Labchart Viewer (version 8.1.14).

### 4.12. Statistical Analyses

All data are reported as mean ± standard error of the mean (SEM). Normality was assessed using Q–Q plots. A two-way ANOVA was then performed to evaluate whether the DOCA–salt intervention and/or the LDN-27219 treatment contributed significantly to group variability. When a significant treatment effect was identified, post hoc comparisons between LDN-27219 and vehicle were made within each group using Sidak’s correction for multiple comparisons. *p*-values < 0.05 were considered significant. All analyses and corresponding graphs were carried out in GraphPad Prism (V. 8.4.3).

## 5. Conclusions

Despite the one-kidney DOCA–salt model not being able to induce sustained kidney damage enough for the generation of kidney fibrosis and the lack of hypertension development, the TG2 inhibitor LDN-27219 showed signs of reducing ECM deposition, elucidating the role of TG2 as a driver in early stages of kidney damage. Moreover, the enhancement of ex vivo resistance artery relaxation shows that in vivo LDN-27219 treatment has beneficial vascular effects. Thus, the blockage of TG2 could be beneficial for both the vascular and the renal systems.

## 6. Patents

Estefano Pinilla and Ulf Simonsen are inventors of patent #EP3607948A1 owned by Aarhus University on the use of compound LDN-27219 and derivatives thereof for use in the treatment of diseases.

## Figures and Tables

**Table 1:** Animal and organ weights, urine volume, urea concentrations and mean arterial blood pressure (MAP).

	DOCA		Sham		*p*-value (Effect of DOCA)
	LDN-27219 (n = 12)	Vehicle (n = 10)	LDN-27219 (n = 9)	Vehicle (n = 10)	
Weight before treatment (g)	23.3 ± 1.8	24.0 ± 1.7	24.1 ± 1.0	23.5 ± 1.2	0.81
Weight after treatment (g)	24.9 ± 2.3	24.5 ± 1.7	24.7 ± 1.4	24.4 ± 1.5	0.87
Heart (mg/g)	5.14 ± 0.15	5.54 ± 0.14	5.02 ± 0.14	4.81 ± 0.11	0.006
Liver (mg/g)	5.74 ± 0.12	5.87 ± 0.17	5.22 ± 0.14	4.86 ± 0.07	<0.0001
Kidney (mg/g)	1.08 ± 0.03	1.08 ± 0.03	0.88 ± 0.05	0.85 ± 0.01	<0.0001
Spleen (mg/g)	5.54 ± 0.82	3.94 ± 0.54	2.91 ± 0.30	2.77 ± 0.38	0.006
24 h urine volume (mL)	7.9 ± 2.7 *	12.3 ± 5.9	1.5 ± 0.7	1.6 ± 1.6	<0.0001
P-urea (mmol/L)	5.7 ± 0.4	5.1 ± 0.8	9.4 ± 1.9	9.2 ± 1.4	0.0003
MAP at inclusion (mmHg)	77.6 ± 2.3	84.8 ± 3.5	87.8 ± 3.9	80.9 ± 3.8	0.38
MAP before treatment (mmHg)	82.7 ± 3.2	88.1 ± 2.3	91.4 ± 4.8	82.1 ± 4.1	0.70
MAP after treatment (mmHg)	80.4 ± 7.6	85.2 ± 3.1	83.3 ± 4.3	83.6 ± 2.5	0.90

Data are mean ± SEM. The column with *p*-values represents the effect of DOCA based on a 2-way ANOVA. * *p* < 0.05, LDN-27219 compared to vehicle.

**Table 2:** Characteristics of the isolated mesenteric small arteries.

	DOCA		Sham	
	LDN-27219 (n = 12)	Vehicle (n = 10)	LDN-27219 (n = 9)	Vehicle (n = 8)
Internal diameter (µm)	205 ± 27	202 ± 41	191 ± 54	235 ± 58
Contraction to KPSS (mN)	5.1 ± 0.7	4.5 ± 0.5	4.6 ± 0.5	5.3 ± 0.6
Contraction to KPSS (mN) repeated	4.5 ± 0.5	4.1 ± 0.5	4.3 ± 0.6	5.1 ± 0.7
Contraction before ACh (mN)	3.6 ± 0.4	3.8 ± 0.6	4.3 ± 0.6	5.0 ± 0.5
Contraction before SNP (mN)	2.9 ± 0.3	3.0 ± 0.4	2.7 ± 0.5	3.7 ± 0.5

Data are mean ± SEM, with n referring to the number of animals in each group. ACh, acetylcholine; SNP, sodium nitroprusside; KPSS, physiological saline solution.

## Data Availability

Data will be available upon reasonable request to the corresponding author.

## Supplementary Materials

The following supporting information can be downloaded at: https://www.mdpi.com/article/10.3390/ijms26125724/s1.

## Abbreviations

Ach	acetylcholine
BP	blood pressure
BPA	biotinamide pentylamine
DOCA	deoxycorticosterone acetate
MAP	mean arterial pressure
NA	noradrenaline
NO	nitric oxide
PSS	physiological saline solution
SNP	sodium nitroprusside
TGF-β	transforming growth factor beta
TG2	transglutaminase 2
