# Supplementary material for: Renal and Vascular Effects of the Allosteric Transglutaminase 2 Modulator LDN-27219 in One-Kidney DOCA–Salt Mice
Source: Int J Mol Sci. 2025 Jun 14;26(12):5724. doi: 10.3390/ijms26125724 (PMC12192933; doi:10.3390/ijms26125724)
Supplement: Supplementary file 1 [file ijms-26-05724-s001.zip › ijms-3636664-supplementary.pdf]

Supplementary material

Supplementary Table S1

Supplementary material

Supplementary Table S1. Scoring of animal welfare.

**Parameter**	**Method**	**Result**
Movement	Voluntarily movement, When cupped in hand and put gently back.	0: Going quickly back to normal behavior (for example drinking, seeking shelter)<br><br>1: Walking abnormal or slower than expected.<br><br>2: Barely moving, not moving.
Water or food intake: (to check for possible pain when stretching for food or water)	Watching the mouse drink or eat	0: Mouse is comfortable reaching up to drink or eat.<br><br>1: Mouse is not reaching up for water or food.
Fur	Grooming	0: Well groomed, normal appearance.<br><br>1: Hair not well groomed / ruffed.<br><br>2: Dirt in face or eyes, piloerection.
Wound	Inspection of wound.	0: No swelling, no exudate, no major redness. Most sutures in place.<br><br>1: More than one suture missing<br><br>2: Clear signs of infection/inflammation, clear indication the mouse tries to gnaw on wound and sutures.
Behavior	Response to the handler When touched.	0: Normal flight response when brushed on back with a finger (going away, seeking shelter)<br><br>2: Abnormal behavior (not moving, trying to bite)
If together with another mouse signs of fighting	Biting marks, fighting	0: Mice comfortable touching each other without aggressive behavior.<br><br>1: Biting marks. Fighting in front of caretaker.

Supplementary Table S2

Supplementary material

Supplementary Table S2. Sequences of the primers and probes used for qPCR.

Target	Forward primer	Reverse primer	Probe
Tgm2	GAGGCAGGGACCAAGGCC	ATCGGCTGGGCACCAGGCATT	TCCAGCTTTGTGCTGGGCCACTTCAT
Fibronectin	AATGGAAAAGGGGAATGGAC	CTCGGTTGTCCTTCTTGCTC	TAGGCGAAGGCAATGGACGCAT
Collagen 1α1	GCTGACCTTCCTGCGCCTAAT	GCGGGAGGTCTTGGTGGTTTT	AGTGATAGGTGATGTTCTGGGAGG
Collagen 3α1	ATTCTCCCCAATTCGACTCATA	ACTCTCCATCCTTTCCAGCA	CCACCCATTCCTCCCACTCCAGACTT
αSMA	GACAGCTATGTGGGGGATGA	AGAGGCATAGAGGGACAGCA	ATCCTGACGCTGAAGTATCCGAT
TGFβ	ACCAAAGACATCTCACACAGTATA	GCTGAATCGAAAGCCCTGTA	TGACCCCCACTGATACGCCTGA
Gadph	CACGGCAAATTCAACGGCACAG	AGACTCCACGACATACTCAGCACC	AGCTTGTCATCAACGGGAAGCCCAT CACCA

Tgm2, transglutaminase 2; αSMA, α-smooth muscle actin; TGFβ, tumor growth factor β; Gadph, glyceraldehyde-3-phosphate dehydrogenase.

Supplementary Figure S1

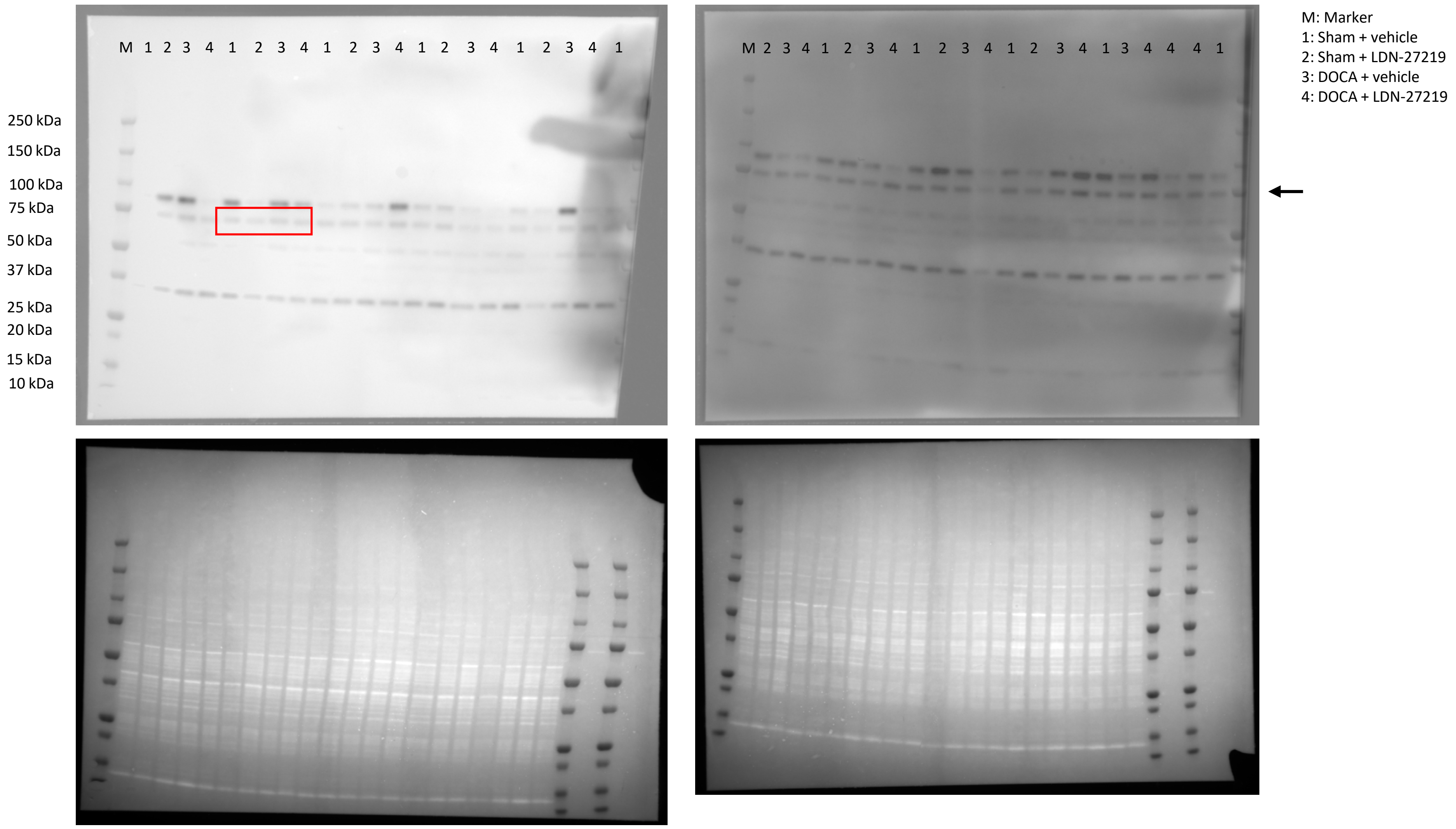

Original TG2 blots

Supplementary Figure S2

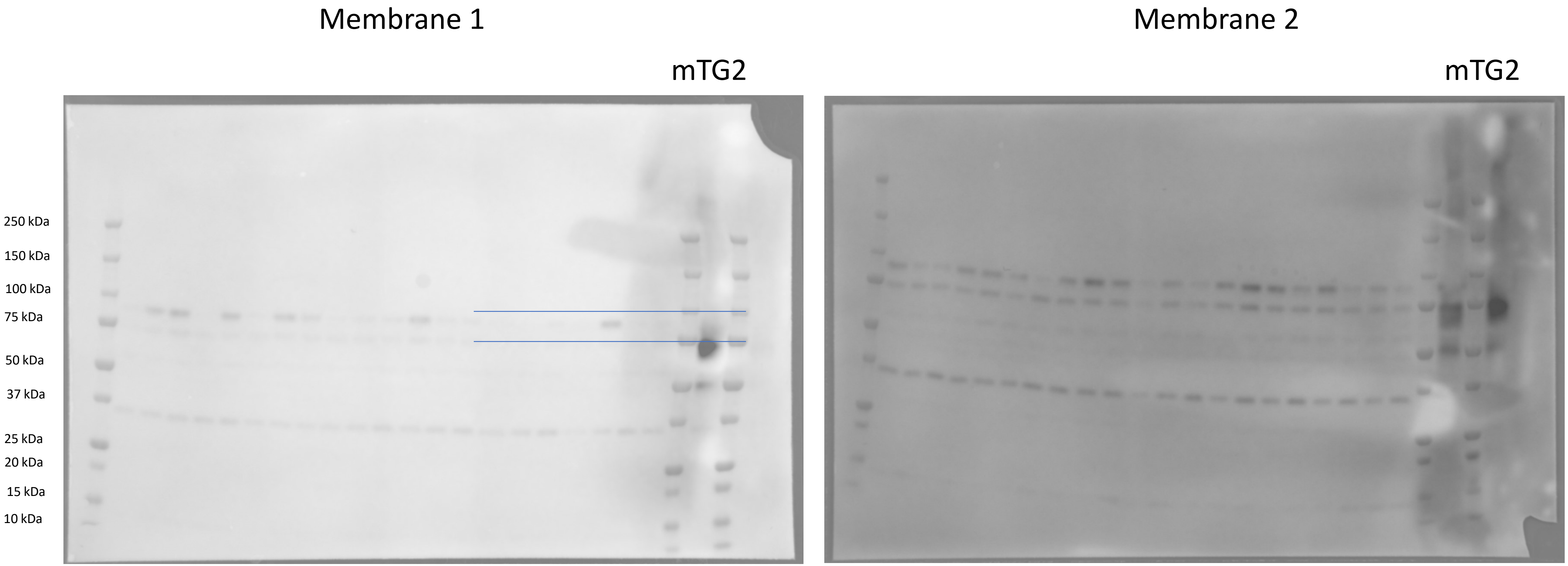

Full blots of TG2 including the positive control with mouse TG2 (mTG2) from the membranes shown in supplementary figure 1.

Supplementary Figure S3

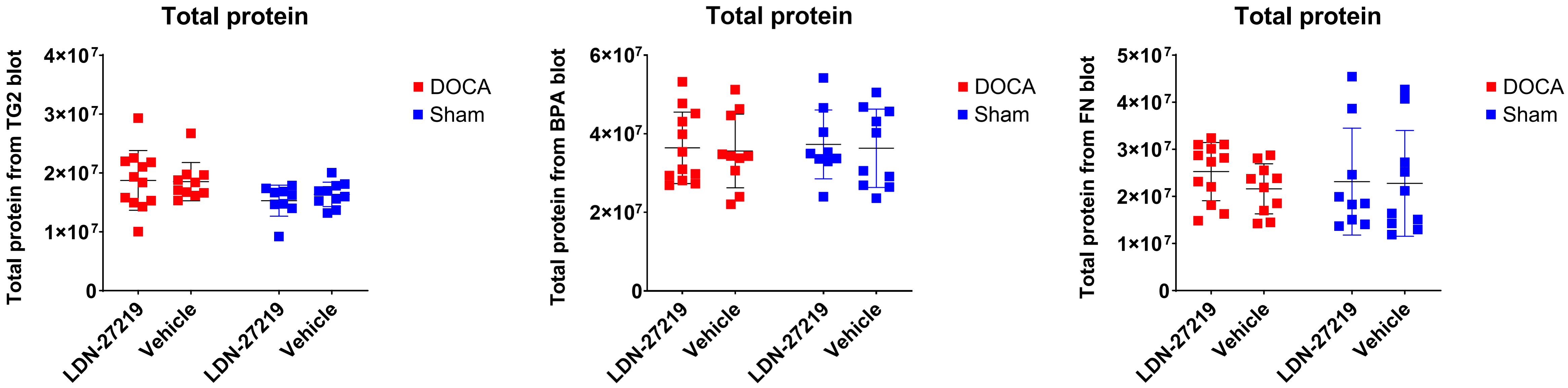

Total protein quantification from TG2, BPA and fibronectin (FN) Western blots.

Supplementary Table S3

Cq values for PCR experiments

Sample name	TG2	Fibronectin	Collagen 1α1	Collagen 3α1	αSMA	TGFβ1	Gapdh
1	24,99	23,36	27,32	28,64	24,22	26,05	16,01
2	24,21	22,98	26,21	27,11	23,65	25,44	15,43
3	24,21	21,86	24,69	25,94	24,13	26,23	16,58
4	25,42	23,06	26,48	27,36	24,12	26,22	15,93
5	26,26	22,91	27	27,35	24,57	25,12	15,63
6	25,52	21,84	24,95	25,98	22,97	25,15	15,5
7	24,42	21,31	24,63	25,33	23,1	24,72	15,6
8	24,21	21,88	24,66	25,24	22,97	24,95	15,48
9	24,14	21,34	24,46	25,61	22,77	24,16	15,83
10	24,42	21,93	25,63	26,05	23,53	26,45	16,31
11	23,27	17,7	19,05	20,36	19,43	22,24	15,81
12	24,47	21,86	24,71	26,25	24,36	25,74	16,21
13	23,64	19,41	22,37	23,45	21,68	23,22	15,62
14	24,26	23,1	26,44	27,04	24,25	26,38	15,56
15	24,71	22,87	25,42	26,57	23,95	25,41	16,28
16	24,93	23,5	27,33	27,67	24,64	25,78	16,32
17	26,11	22,41	25,49	25,98	24,59	24,64	15,89
18	23,8	19,68	21,95	22,97	22,94	23,42	15,48
19	24,44	22,92	26,95	27,5	24,03	25,76	15,39
20	24,37	21,09	24,27	24,56	23,04	24,42	15,29
21	23,98	21,4	24,56	24,61	23,68	23,62	15,23
22	25,95	21,31	24,8	25,3	23,99	24,07	15,81
23	24,2	22	25,47	25,98	23,8	25,6	15,68
24	24,88	22,27	26,68	27	23,48	25,66	16,53
25	24,83	21,44	24,17	26,18	23,93	25,92	15,99
26	23,99	21,98	24,49	25,69	23,33	25,3	15,53
27	22,4	20,07	22,25	23,59	23,1	24,05	15,3
28	24,37	23,17	25,87	26,44	24,16	26,61	15,97
29	25,04	22,48	25,73	26,71	24,33	26,7	15,48
30	23,46	20,87	21,97	24,2	22,51	25,77	15,08
31	23,09	18,87	20,92	21,57	21,69	23,8	16,11
32	23,49	21,87	25,04	25,43	23,15	25,96	15,64
33	23,27	21,05	24,87	25,58	24,05	25,82	16,31
34	23,46	21,4	25,31	25,4	23,72	25,94	15,88
35	24,82	23,66	28,14	28,86	25,03	26	16,22
36	24,37	22,93	27,08	28,08	24,6	26,4	16,28
37	25,17	23,28	26,82	28,61	24,37	26,37	16,02
38	26	21,95	25,57	26,51	24,68	25,93	16,45
39	23,8	21,57	24,7	25,56	23,58	25,44	15,61
40	23,6	21,44	24,74	25,87	22,87	25,4	14,96
NTC	No Cq	No Cq	38	No Cq	No Cq	No Cq	No Cq

**Sham vehicle:**	**Sham LDN-27219:**	**DOCA vehicle:**	**DOCA LDN-27219:**
1	3	6	7
2	4	9	8
5	13	17	10
12	15	22	11
14	16	24	20
18	19	32	21
25	28	33	23
26	36	38	29
27	37	40	30
35			31
			34
			39

Supplementary Figure S4

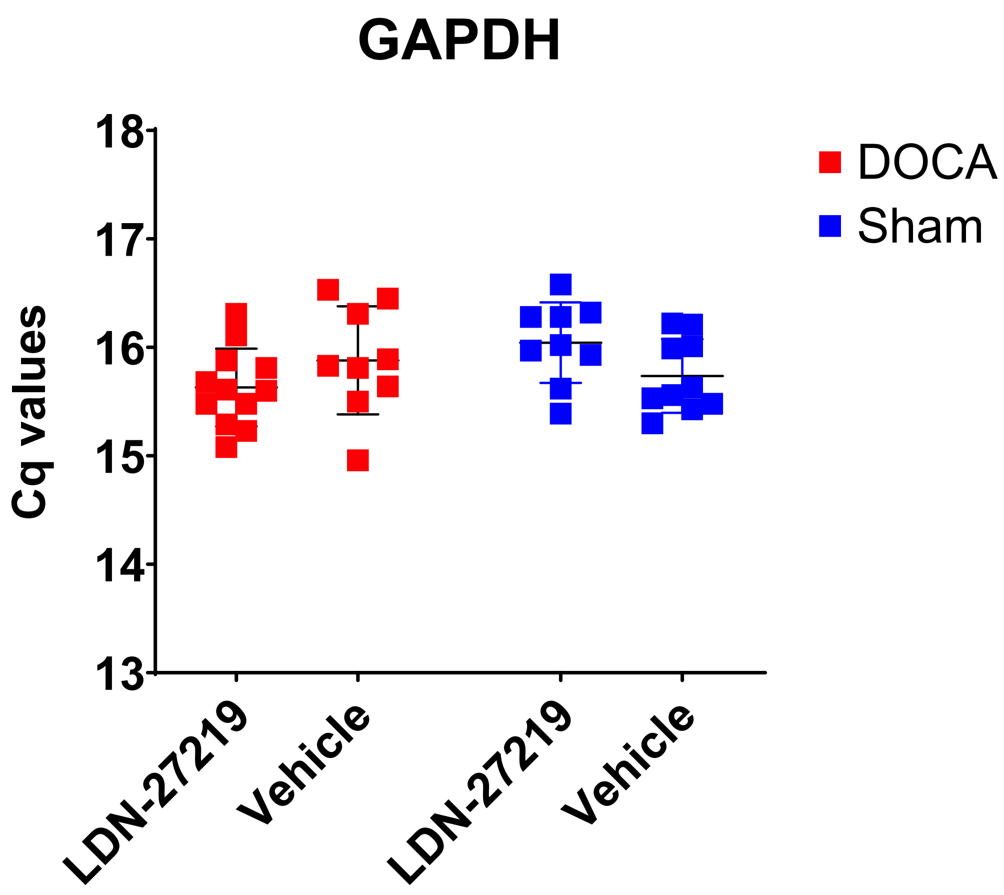

Cq values from GAPDH PCR experiments.

Supplementary Figure S5

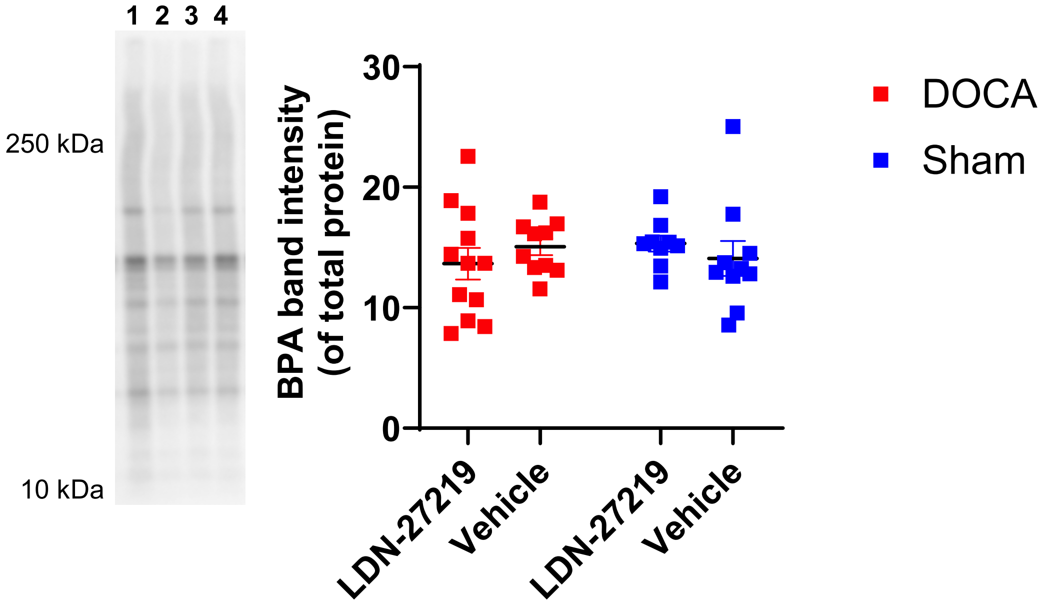

Quantification of the BPA incorporation in kidney samples of DOCA mice treated with LDN-27219 (n=12) or vehicle (n=7) and in sham mice treated with LDN-27219 (n=9) or vehicle (n=10), with a representative blot. Lane 1: sham + vehicle, lane 2: sham + LDN-27219, lane 3: DOCA + vehicle, lane 4: DOCA + LDN-27219. Data are expressed as mean ± SEM.

Supplementary Figure S6

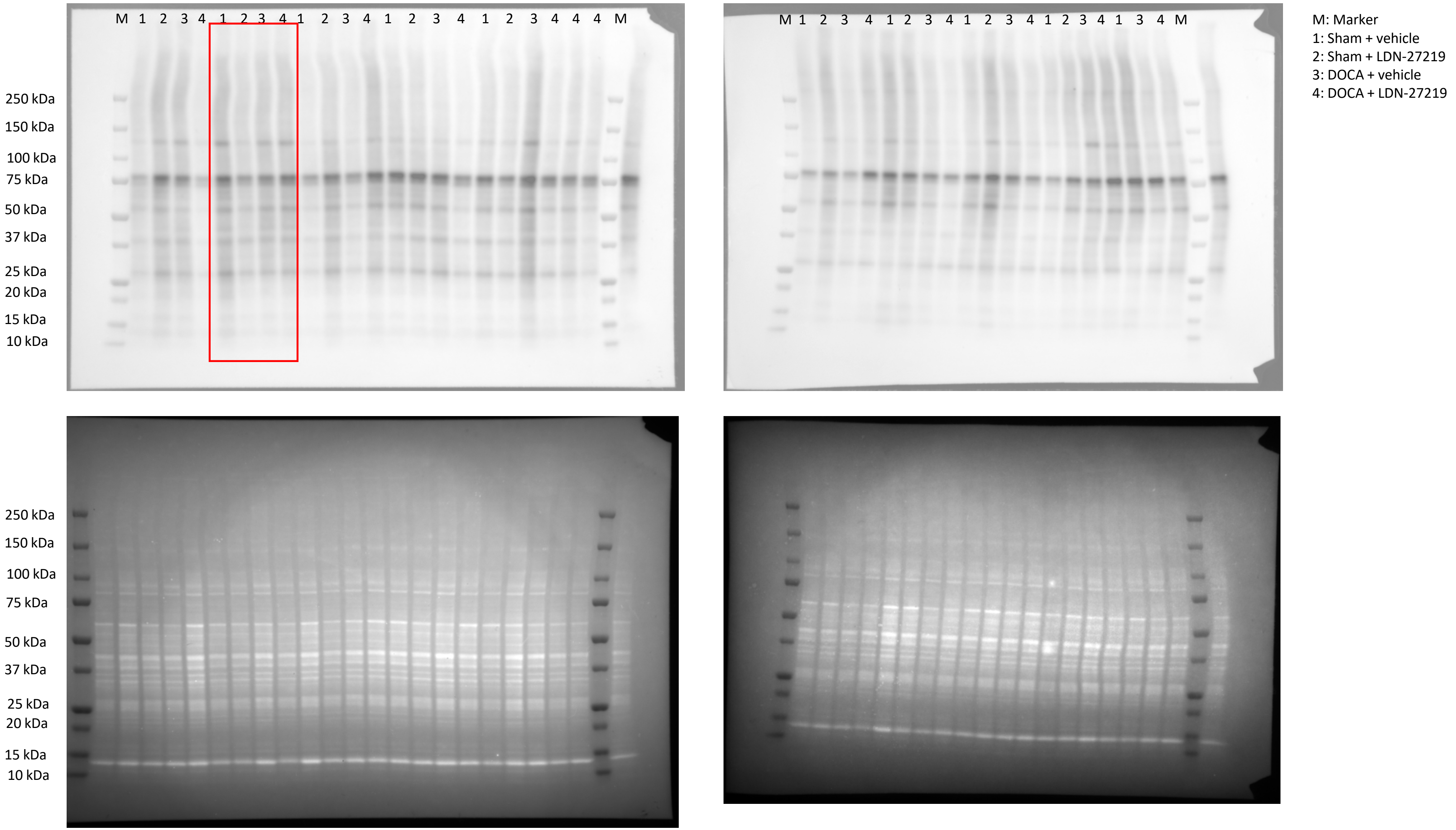

Original BPA blots

Supplementary Figure S7

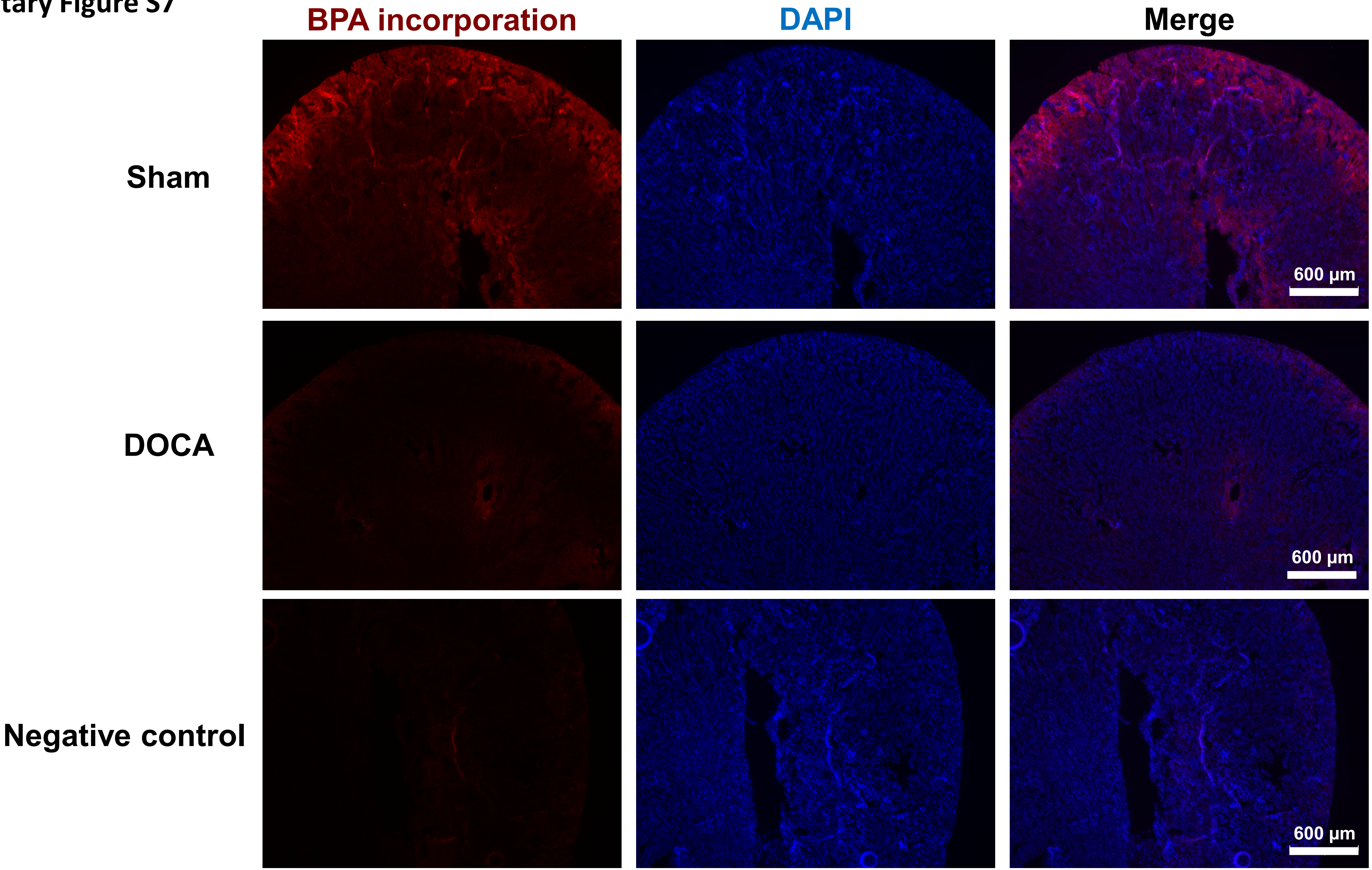

Legend for supplementary Figure S7:

Staining of BPA incorporation (red) of sham and DOCA kidneys counterstained with DAPI (blue). BPA incorporation was found in the superficial cortical area of the kidney, indicating a lack or permeability of the compound. Scale bar corresponds to 600 µm. The pictures show representative examples.

Supplementary Figure S8

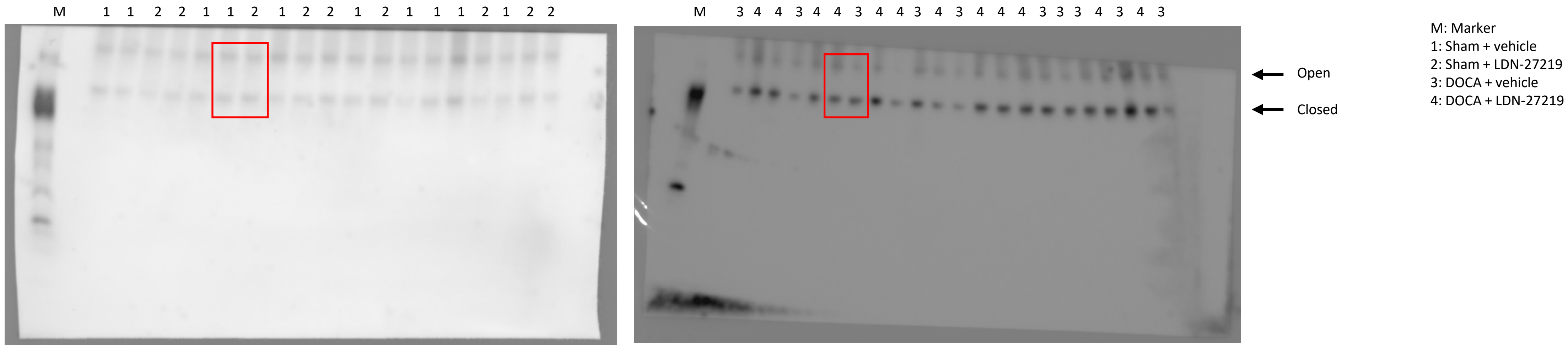

Original native gel blots

Supplementary Figure S9

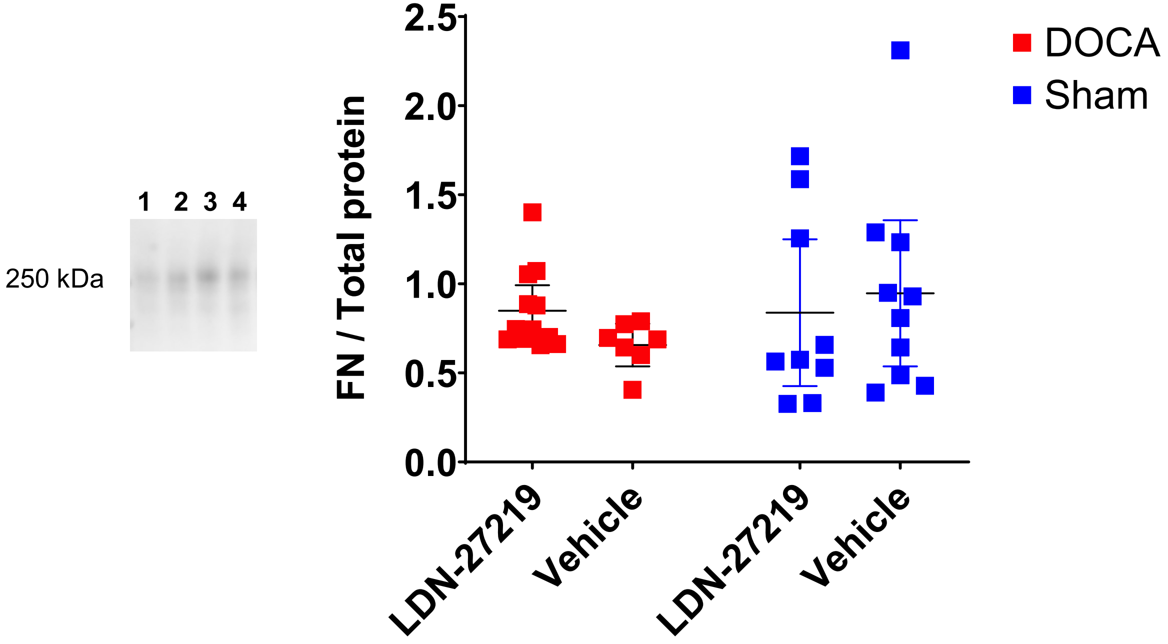

Kidney fibronectin (FN) protein expression in DOCA mice treated with LDN-27219 (n=12) or vehicle (n=7) and in sham mice treated with LDN-27219 (n=9) or vehicle (n=10) with a representative blot. Lane 1: sham + vehicle, lane 2: sham + LDN-27219, lane 3: DOCA + vehicle, lane 4: DOCA + LDN-27219. Data are expressed as mean ± SEM.

Supplementary Figure S10

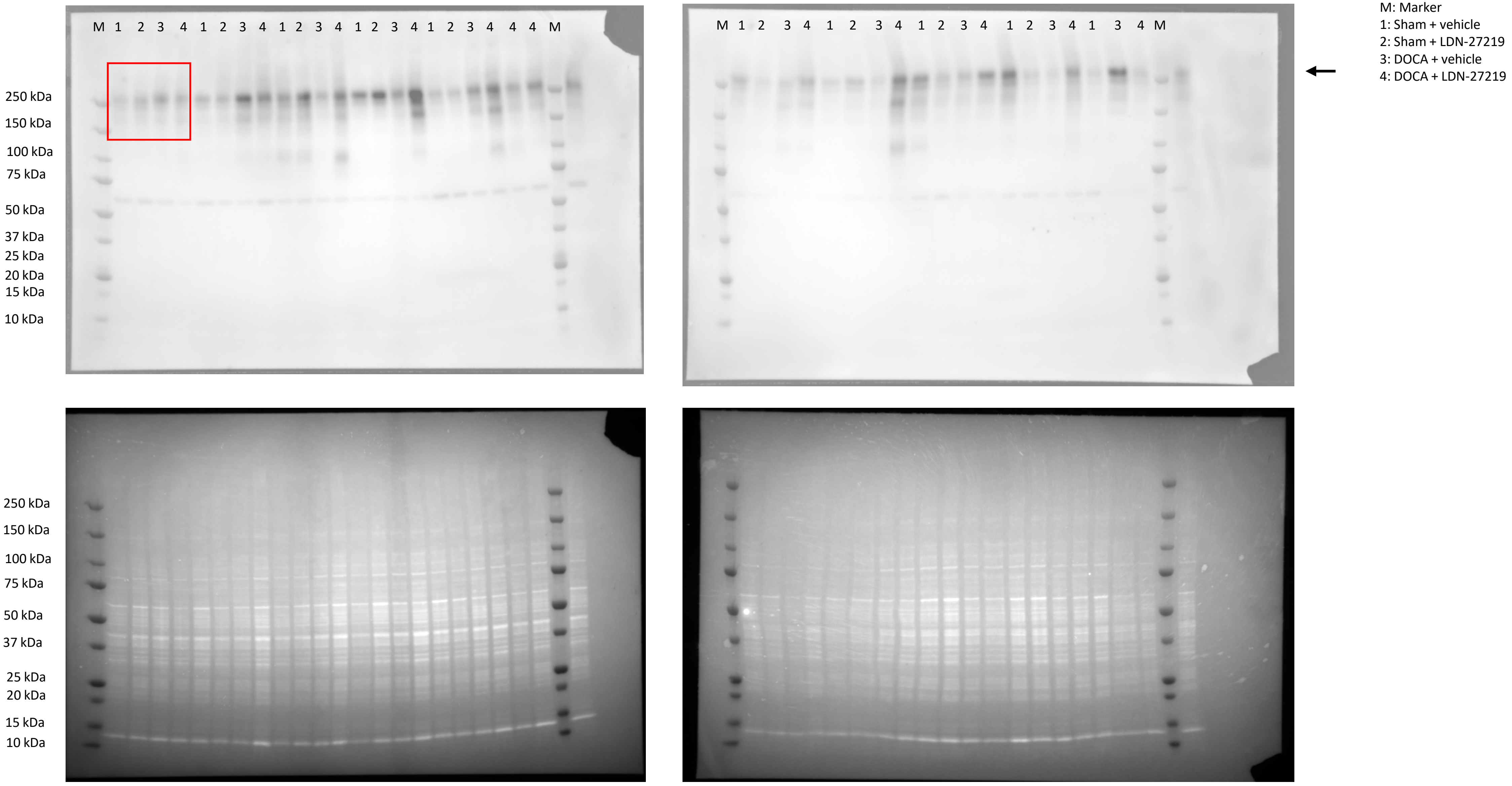

Original fibronectin blots
